# Infantile Hemangioma in Infancy: A Case Study on the Natural Course and Therapeutic Management

**DOI:** 10.7759/cureus.63156

**Published:** 2024-06-25

**Authors:** Mansoor Gullabzada, Yesenia Brito, Ana I Gonzalez, Dawit Zena, Mohamed N Jabri

**Affiliations:** 1 Medicine, Saint James School of Medicine, Arnos Vale, VCT; 2 Surgery, St. George's University School of Medicine, True Blue, GRD; 3 Medicine, St. George's University School of Medicine, True Blue, GRD; 4 Pediatrics, Jabri Medical Ltd., Bloomingdale, USA; 5 Pediatrics, Alexian Brothers Hospital, Elk Grove Village, USA

**Keywords:** cutaneous hemangioma, pediatric dermatology, benign vascular tumors, strawberry hemangioma, infantile hemangioma

## Abstract

Infantile hemangiomas (IHs) are common benign vascular tumors that affect infants. In this case report, we detail the natural course of an IH in an infant monitored over four months without medical intervention, illustrating the benign progression and potential for spontaneous stabilization of such lesions. The aim was to observe changes in the size and morphology of the hemangioma, alongside the infant's overall health and developmental milestones, through regular clinical assessments. This case presented a challenge as the patient's parents lacked English fluency, lacked healthcare access, and had low socioeconomic status. It highlights the importance of individualized patient care, advocating for careful observation and restraint in the application of pharmacological treatments when clinically unnecessary. The report contributes to existing pediatric dermatology knowledge by emphasizing the natural benign behavior of IH and the need for a balanced approach to treatment decisions, ensuring safe and favorable long-term outcomes for patients.

## Introduction

Infantile hemangiomas (IHs), also known as strawberry hemangiomas, are benign vascular tumors characterized by bright red papules, nodules, or plaque raised above the skin [1]. These lesions can be either congenital (30%) or more frequently infantile (70%), which emerge between the second and eighth week of life [2]. IHs are distinguished from other vascular tumors by their rapid endothelial cell proliferation followed by gradual involution. The incidence of IHs has yet to be established. Some studies suggest 4%-5% [3], whereas others have indicated up to 10% of Caucasian infants [4]. The risk factors include female sex, white non-Hispanic infants, preterm infants with low birth weight, older maternal age, placenta previa, preeclampsia, and other placental anomalies [5].

The proliferation course of IH raises considerations for intervention, particularly in instances where they pose a risk to critical functions or bear the potential for disfigurement. While the standard of care includes the use of nonselective beta-blockers like propranolol and topical timolol for their efficacy in facilitating regression, the approach to each case must be individualized, considering the lesion's size, location, and the patient's overall health [6]. Corticosteroids serve as an alternative in cases where beta-blocker therapy is contraindicated or ineffective, and surgical options are considered when medical management fails or the hemangioma threatens the patient's quality of life [7].

In preparing to discuss a specific case of IH in an infant, this introduction sets the stage for exploring the diagnostic journey, the decision-making process surrounding treatment, and the outcomes achieved. This case presented a challenge as the patient's parents lacked fluency in English and healthcare access and had low socioeconomic status. By focusing on a singular case, we aim to contribute to the broader understanding of how these common but complex lesions can be managed effectively and safely in the pediatric population, underscoring the importance of tailored, patient-centric care strategies.

## Case presentation

The patient, a female infant of a Hispanic background, first presented to the outpatient clinic at five days old for a newborn screening. She was born via natural delivery at 38 weeks with no complications during pregnancy or birth. At this initial visit, she weighed 7 lbs, measured 19.2 inches in height, and had a head circumference of 13.1 inches. The baby was doing well, feeding every two to three hours for 15 minutes per breast, producing 8-10 wet diapers and three to four stools daily, all yellowish and of watery consistency. She had passed the hearing test and received vitamin K and the first dose of the hepatitis B vaccine at the hospital. The parents reported no concerns, and the baby appeared to be in good health, meeting all expected newborn milestones.

**Figure 1:**
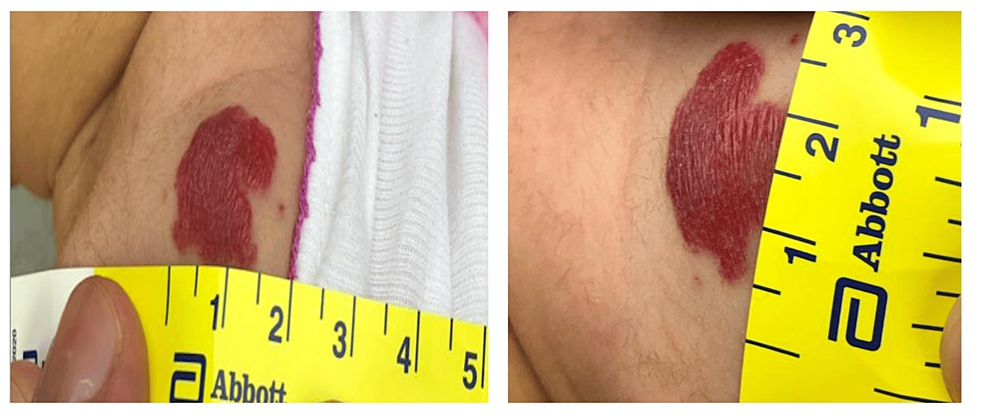
Infantile hemangiomas (IHs) on the back of the left shoulder, measuring 1.5 x 2 cm. The patient at one month old.

The concern regarding the hemangioma arose during a follow-up visit when the patient was one month old. The mother reported noticing a red lesion on the back of the left shoulder while bathing her daughter (Figure 1). This lesion was not present at birth but developed weeks after. Upon examination on January 9, 2024, the hemangioma measured 1.5 x 2 cm with slight blanching on the stretch. No size, color, or other associated features had changed as it was first noticed. The infant showed no discomfort or irritation from the hemangioma, which did not affect her feeding or sleeping patterns. The mother reported no use of specific products or treatments for the hemangioma at home, and the baby had not been exposed to any illnesses or medications since birth.

Throughout the patient's development, the mother reported that the baby had grown and developed normally since birth. There were no concerns regarding milestones, feeding, or weight gain. The baby continued to be exclusively breastfed, showing average growth and developmental progress. By the fourth-month follow-up visit, the hemangioma's characteristics remained unchanged. Her overall health was reported to be good, with no developmental concerns.

This case of IH exemplifies the benign and typically uncomplicated nature of such vascular tumors in infants. Most infantile hemangiomas do not require intervention; instead, parents need to be educated on the natural course of the lesion, treatment options, and anticipatory guidelines. This case presented a challenge as the patient's parents, who lacked fluency in English, had limited healthcare access and low socioeconomic status. The parents were educated on the diagnosis through a Spanish translator upon the patient’s initial presentation. They were provided with a referral to take the patient to a dermatologist for further evaluation. However, during the subsequent visit, the parents reported that they had yet to visit the dermatologist because of financial limitations and declined the offer of a specialist referral. Recognizing this barrier, the parents were informed to set up regular follow-up appointments to monitor the progression of the lesion. Moreover, they were advised to monitor the lesion for bleeding, ulceration, and infection, as such complications may require treatment.

In resource-poor settings, specialty consultation and access to healthcare are not always available. In such cases, primary care physicians can use mainstream treatments such as steroids or nonselective beta-blockers with careful monitoring and parental education.

## Discussion

Pathophysiology

Vascular lesions in children are of two main types: vascular tumors and vascular malformation. These lesions are classified based on Mulliken and Glowacki’s 1982 classification that considers histology, biological, and clinical manifestations. Generally, vascular malformation is a disorder of vessel morphogenesis. In contrast, in particular infantile hemangiomas, vascular tumors are characterized by periods of rapid angiogenesis followed by regression and replacement with fibrofatty tissue. Table 1 provides examples of vascular lesions [8]. The exact pathophysiology of infantile hemangioma has yet to be clearly understood. However, several hypotheses have been established.

**Table 1:** Classification of vascular lesions.

Vascular Tumors	Vascular Malformation
Congenital hemangioma	Arteriovenous malformations
Infantile hemangioma	Capillary malformations
Kaposiform hemangioendothelioma	Lymphatic malformations
Tufted angioma	Venous malformation

Proliferation and involution

One hypothesis suggests that hemangioma endothelial cells arise from disrupted placenta tissue embedded in fetal soft tissue during gestation or birth. The expression of GLUT1 and placenta-associated vascular antigens in endothelial cells of infantile hemangiomas and placental tissue has supported this. Thus, indicating a common genetic origin. Moreover, it has been proposed that local or utero hypoxia may be the initiating factor for the development of hemangiomas. This is supported by findings of multiple hypoxia-inducible factor 1-alpha being expressed in a proliferating hemangioma. Such factors include endothelial cell markers (e.g., CD34 and CD31), insulin-like growth factor-2 (IGF-2), basic fibroblast growth factor (bFGF, FGF-2), vascular endothelial growth factor (VEGF), proliferating cell nuclear antigen, and type IV collagenase I [9]. IH's spontaneous involution begins at the end of the first year and continues over a variable number of years. This phase is characterized by increased apoptosis and histologic fibrosis of capillary lumina. An increased number of tissue metalloproteinases, upregulation of interferon-induced genes, and decreased quantities of bFGF in urine have been observed in this phase [10].

Clinical features and associated conditions

Hemangiomas can be superficial, deep, or compound. Superficial hemangiomas are most common and present as bright red papules, nodules, or plaque raised above the skin. Deep hemangioma is a protrusion with an overlying bluish hue with or without telangiectasia. Compound hemangiomas have both deep and superficial presentations. Another subclassification for hemangioma is focal or segmental. Focal hemangiomas are unilocular lesions that are spatially confined, whereas segmental hemangiomas are diffuse, plaque-like, and are more likely to be associated with complications. Segmental hemangiomas have been associated with other conditions such as posterior fossa malformations, hemangioma of the cervicofacial region, arterial anomalies, cardiac anomalies, eye anomalies (PHACE) syndrome and Sternal or abdominal clefting, spinal dysraphism, anogenital, cutaneous, renal and urologic anomalies, associated with an angioma of lumbosacral localization (SACRAL), and perineal hemangioma, external genitalia malformations, lipomyelomeningocele, vesicorenal abnormalities, imperforate anus, and skin tag (PELVIS) syndrome. Finally, spinal dysraphism, which is a midline abnormality because of defective fusion of the neural tube leading to spinal abnormalities. It has been demonstrated that cutaneous infantile hemangiomas of the lumbosacral region have a high risk of association with spinal dysraphism [7,11].

Management and complications

Management of IH depends on several factors such as type of hemangioma; stage of lesion, location, and extent; associated systemic involvement; and psychosocial distress of parent or child. Generally, the majority of IHs do not require intervention. However, IH that poses a risk to the face, perineum, or airway requires intervention. The two main treatments of IH are systemic steroids (prednisolone) and nonselective beta-blockers (propranolol or topical timolol). Before initiation of treatment, contraindications to steroids should be addressed, such as primary immunodeficiency disease and active infection. For nonselective beta-blockers, contraindications such as bronchial asthma, heart failure, heart block, and allergy to medication must be addressed. Treatment options such as vincristine, cyclophosphamide, and interferon-alpha are reserved for unresponsiveness to mainstream drug therapy and life-threatening hemangioma [12].

Steroids (Prednisolone)

Steroids are most effective in the proliferative phase. The recommended dosage is 2-4 mg/kg/day, which should be continued until the involution of the hemangioma, followed by a tapering period. Side effects such as hypertension, irritability, fussiness, and insomnia have been reported. These symptoms typically resolve after the cessation of the drug regimen [12,13].

Non-selective Beta-Blockers (Propranolol and Timolol)

Propranolol's β2 inhibitory effect decreases nitric oxide, a vasodilator molecule, resulting in decreased blood supply to the hemangioma. Moreover, it has a downregulation effect on VEGF and bFGF, inhibiting angiogenesis. A 1-3 mg/kg/day dosage has been recommended. Side effects such as decreased heart rate and blood pressure, hypoglycemia, and pulmonary symptoms have been observed [3,12-13]. To minimize the systemic side effects caused by oral propranolol, topical timolol has been proven to be as effective for superficial lesions. Timolol's mechanism of action includes reducing blood flow through hemangioma by blocking β-adrenergic receptors, inhibiting growth factor responsible for the proliferative phase, and aiding in apoptosis [14]. The recommended dosage is to apply timolol 0.5% solution one drop twice daily. Timolol is well tolerated and a safer treatment option with the best response in superficial IHs [12].

## Conclusions

IHs are benign vascular tumors, characterized by bright red papules, nodules, or plaque raised above the skin that emerges between the second and eighth weeks of life. The pathophysiology is yet to be clearly understood. Most IHs do not require intervention; instead, parents need to be educated on the natural course of the lesion, treatment options, and anticipatory guidelines. This case presented a challenge as the patient's parents, who lacked fluency in English, had limited healthcare access and a low socioeconomic status. In resource-poor settings, primary care physicians can use mainstream treatments such as steroids or nonselective beta-blockers with careful monitoring and parental education.
